# Dopamine Transporter Genotype Dependent Effects of Apomorphine on Cold Pain Tolerance in Healthy Volunteers

**DOI:** 10.1371/journal.pone.0063808

**Published:** 2013-05-21

**Authors:** Roi Treister, Dorit Pud, Richard P. Ebstein, Elon Eisenberg

**Affiliations:** 1 The Rappaport Faculty of Medicine, Technion - Israel Institute of Technology, Haifa, Israel; 2 Faculty of Social Welfare and Health Sciences, University of Haifa, Haifa, Israel; 3 Institute of Pain Medicine, Rambam Health Care Campus, Haifa, Israel; 4 Psychology Department, Hebrew University, Jerusalem, Israel; Radboud University, The Netherlands

## Abstract

The aims of this study were to assess the effects of the dopamine agonist apomorphine on experimental pain models in healthy subjects and to explore the possible association between these effects and a common polymorphism within the dopamine transporter gene. Healthy volunteers (n = 105) participated in this randomized double-blind, placebo-controlled, cross-over trial. Heat pain threshold and intensity, cold pain threshold, and the response to tonic cold pain (latency, intensity, and tolerance) were evaluated before and for up to 120 min after the administration of 1.5 mg apomorphine/placebo. A polymorphism (3′-UTR 40-bp VNTR) within the dopamine transporter gene (*SLC6A3*) was investigated. Apomorphine had an effect only on tolerance to cold pain, which consisted of an initial decrease and a subsequent increase in tolerance. An association was found between the enhancing effect of apomorphine on pain tolerance (120 min after its administration) and the *DAT-1* polymorphism. Subjects with two copies of the 10-allele demonstrated significantly greater tolerance prolongation than the 9-allele homozygote carriers and the heterozygote carriers (p = 0.007 and p = 0.003 in comparison to the placebo, respectively). In conclusion, apomorphine administration produced a decrease followed by a genetically associated increase in cold pain tolerance.

## Introduction

While the role of norepinephrine, serotonin and opioids in pain perception is well established, the evidence suggesting that dopamine might also be involved in these processes is relatively limited. In rodents, the excitation of dopaminergic transmission has been shown to induce analgesia [1]. In humans, increased sensitivity to pain has been demonstrated in Parkinson's patients as compared to healthy controls [2]. At the same time, painful clinical conditions, such as burning mouth syndrome, fibromyalgia, and restless leg syndrome, are suggested to be linked to abnormalities in dopaminergic neurotransmission [3]–[5].

Additional support for this hypothesis emerges from genetic studies. In a recent study conducted in our laboratory on a large cohort of healthy subjects and their parents, a transmission disequilibrium test revealed associations between the dopamine transporter gene (*DAT-1*), and to a lesser degree between the monoamine oxidase-A gene (*MAO-A*), and the enhanced ability to tolerate experimental cold pain [6]. At the same time, thermal pain threshold and intensity showed no associations with the studied candidate gene polymorphisms. Other reports showing associations between functional polymorphisms in the genes Catechol-O-Methyltransferase (*COMT*) [7], [8], Dopamine Receptor D4 (*DRD4*) [9], [10], and *MAO-A* [11] and pain phenotypes are also in line with our findings, suggesting that a genetic predisposition to dopaminergic activity may be related to pain sensitivity.

Despite this body of evidence, there is no firm concept regarding the exact role of the dopaminergic system in pain processing [12]. Further studies examining the effects of dopamine on experimental pain models in healthy humans are therefore clearly needed. Accordingly, the first aim of the present study was to test whether the administration of a dopamine agonist will affect the response to experimental thermal pain in healthy humans. A second aim of the study was to explore the possible association between the dopamine transporter gene (SLC6A3, DAT-1) 3′-UTR 40-bp variable number tandem repeat (VNTR) polymorphism and the response to the dopamine agonist. The DAT-1 encodes a sodium-dependent dopamine transporter, which regulates extra-cellular dopamine by re-uptaking the neurotransmitter from the synapse to the pre-synaptic neuron. Extracellular dopamine governs, in part, dopamine receptors availability, which in turn, determines the response to apomorphine administration. Hence, it is reasonable to hypothesis that a genotype which is associated with enhanced DAT-1function could result in deficiency of dopamine at synapses and make subjects more responsive to apomorphine administration, or vice versa.

## Methods

### Ethics statement

The study was approved by both national (The Israeli national ethics Committee) and local (Rambam health care campus) ethics Committees, and a written informed consent was obtained from all participants prior to the beginning of the experiment. The study was registered in the ClinicalTrials.gov Protocol Registration System (registration number NCT01744964).

### Subjects

Participants were 105 healthy paid volunteers, including 41 women and 64 men, ranging in age from 18 to 36 (mean age ± SD 26.1±3.6). The volunteers were students who were recruited through advertisements posted on notice boards at local universities. Subjects were eligible for enrollment in the study if they were healthy and free from chronic pain of any type, did not use any medications other than oral contraceptives, and were able to understand the purpose and instructions of the study. Exclusion criteria were any type of medical or painful condition, use of medications or recreational drugs, or pregnancy.

### Instruments

Cold and heat pain thresholds were determined with the method of limits on a Medoc TSA-2001 device (Medoc, Ramat Ishai, Israel). A Peltier thermode, size 30×30 mm, was attached to the skin above the thenar eminence. The baseline temperature was set at 32°C and was increased or decreased at a rate of 1°C/s. The stimulator temperature range was 0–50°C. The subjects were instructed to depress a switch when the stimulus was first perceived as painfully hot or cold. Three readings were obtained for each thermal modality (cold and hot), and their averages were determined as the pain threshold scores.

In order to assess heat pain intensity, two heat pain stimuli of 47°C were delivered, starting from 37°C and increasing at a rate of 10°C/s. The stimuli lasted three seconds each and were interspersed by a 12-second interval. During each stimulus, the subjects were asked to note their maximal pain intensity on a numerical pain scale (NPS), ranging from 0 = “no pain” to 100 = “the worst pain one can imagine.” Heat pain intensity was calculated by averaging the two NPS scores.

The cold pressor test (CPT) apparatus (Heto CBN 8–30 Lab equipment, Allerod, Denmark) was used for cold pain measurements. The CPT is a temperature-controlled water bath with a maximum temperature variance of ±0.5°C, which is continuously stirred by a pump. Subjects were asked to place their right hand in the CPT (1°C) in a still position with their fingers spread wide apart. A stopwatch was simultaneously activated, and the subjects were requested to maintain their hand in the cold water for as long as they could. They were instructed to indicate the exact point in time when the cold sensation began to elicit pain. This time until the first perception of pain was defined as the threshold of cold pain, measured in seconds (sec). Immediately after hand withdrawal, the subjects were asked to mark their maximal pain intensity on an NPS from 0 to 100. The latency to spontaneous hand removal was defined as the pain tolerance, measured in seconds (sec). A cut-off time of 180 s was set for safety reasons. The pain tolerance for subjects who did not withdraw their hand for the entire 180 s was recorded as 180 s.

### Pharmacological intervention

Apomorphine and identical-looking placebo (saline) syringes were prepared by a nurse who had no contact with the subjects. Apomorphine is an injectable, potent, short-acting dopamine agonist. It is administered subcutaneously and has a bio-availability of 100% which assures considerable short-lived dopamine excitation [13]. Most of apomorphine adverse effects can be sufficiently reduced with a preparation of domperidone, a peripheral dopamine antagonist. The subjects were instructed to take domperidone (10 mg, oral) three times a day for three days preceding both study conditions. Based on pilot studies, conducted in our laboratory, that were aimed to assess the tolerability and the effects of apomorphine on experimental pain measures in healthy subjects, 1.5 mg apomorphine was determined to be the appropriate dose for this study.

### Genotyping

DNA was extracted from mouthwash samples donated by the subjects using the Master Pure Kit (Epicentre, Madison, WI). The *DAT-1* 3′-UTR 40-bp VNTR polymorphism is a functional polymorphism, which is located in the 30-untranslated region, containing a 40 bp existing in 3–13 copies [14], [15]. It was amplified by a Polymerase Chain Reaction (PCR) procedure (Perkin-Elmer Cetus 9600 thermal cycler), using the following primers: *DAT-1* forward primer 5′-CTT CCT GGA GGT CAC GGC TCA-3′ and *DAT-1* reverse primer 5′-TGT GGT GTA GGG AAC GGC CTG- 3′. The reaction mixture (20 µl) contained 200 µM dNTPs; 0.25 µM primers; 0.5 unit Taq Gold (Perkin-Elmer Life Sciences, Boston, MA, USA); and 20 ng DNA. The amplification procedure included a 5-min pre-start at 95°C and 30 cycling conditions, as follows: 90°C for 30 s; 55°C for 30 s; and 72°C for 90 s. A final extension was performed at 72°C for 5 min. The reaction mixture underwent electrophoresis in a 3% agaroz gel (Ameresco, Ohio, USA) with etidum bromide. Five variants of a 40-bp repeat sequence have been reported: 7 (360 bp); 9 (440 bp); 10 (480 bp); 11 (520 bp); and 13 (600 bp). Gel images were examined by one blinded researcher.

### Study design

The study was designed as a randomized double-blind, placebo-controlled, cross-over trial. A detailed explanation of the study design was given to all subjects, and their written informed consent was obtained. Subjects were then exposed to five batteries of experimental pain, each consisting of the measurement of thermal pain thresholds and heat pain intensity (TSA), as well as cold pain threshold, tolerance and intensity, using the cold pressor test (CPT). Stimuli order within each test battery was fixed, with thermal thresholds performed at the beginning, followed by heat pain intensity and then finally the CPT at the end of each battery. A 9-minute interval between two consecutive tests within each battery was allowed, and therefore the CPT was performed 20 minutes after the initiation of each test battery. Each battery lasted 25 minutes, with a 20-minute break between two test batteries.

**Figure 1:**
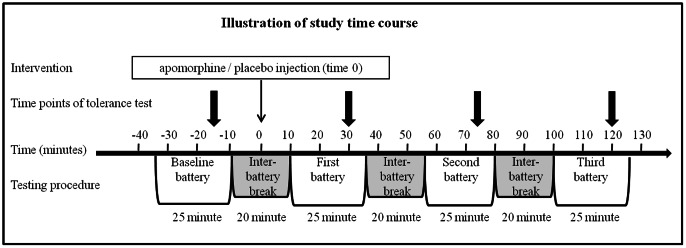
Study design. The black line represents study timeline. Time 0 represents apomorphine/placebo injection. Tolerance was assessed twenty minutes after the initiation of each test battery (at -15, 30, 75 and 120 minutes, represent by black arrows). For a detailed description of study timelines see study design section.

The first battery of pain tests was considered as training, and its results were excluded from all analyses. Twenty minutes later, another battery was conducted and the results were recorded as the baseline measurements. Ten minutes following completion of the baseline measurements, the subjects received either the 1.5 mg subcutaneous apomorphine or an identical placebo (saline) injection. Three additional test batteries were conducted at 10, 55, and 100 minutes after drug administration (figure 1). One week later, a second session was conducted in the same manner with the other treatment (apomorphine or placebo). Randomization of the order of apomorphine and placebo administration was done in blocks of four according to a computer-generated random code.

Following drug administration and just before initiating each test battery, the subjects were asked to self-report the adverse effects experienced prior to the session on a 0–3 scale, ranging from “does not bother at all” to “highly bothers.” The following adverse effects were monitored: sweating, dyspnea, dry mouth, sleepiness, headache, nausea, and confusion.

The experimenter was blinded to both the injected drug (apomorphine/placebo) and to the subjects' adverse effect reports. Subjects were instructed not to discuss their adverse effects with the experimenter unless they were perceived as severe.

### Statistical analyses

Analyses were conducted using the SPSS for Windows Version 19 statistical package (SPSS, Inc., Chicago, IL). Values are presented as means ± standard error of means (SEM) unless specified otherwise. A Shapiro-Wilk W test of normality revealed that the experimental pain measures were not normally distributed. In addition, transformations did not normalize some of the measures. Hence, all analyses were based on non-parametric tests. Given that the adverse effects were measured on an ordinal scale, the adverse effects analysis was non-parametric as well.

Three sets of analyses were performed. The first set was considered as a screening analysis, in which the effect of apomorphine/placebo on the six pain measures was assessed using Friedman tests. Post-hoc Wilcoxon signed ranks tests were applied in order to assess the differences between the specific time points both within and between sessions. The possibility that the findings are the result of a Type I statistical error cannot be discounted. Thus, Bonferroni correction was applied, and the results of the screening analysis were considered significant at the p<0.008 level (0.05/6). The effect sizes were calculated as the absolute value of the Z score divided by the square root of n.

The second set of analyses was aimed to search for associations between genotype and parameters which were significantly affected by apomorphine/placebo (according to the first analysis). Here, the differences in the effect of the apomorphine between genotype groups were assessed using the Kruskal Wallis Test with post hoc Mann-Whitney test. The effect of the apomorphine/placebo was calculated as changes from baseline. In other words, the drug effects were calculated by subtracting the baseline values from the values recorded at each time point. In addition, a complementary comparison between the effects of the apomorphine and the placebo was calculated. This was done by subtracting the difference in the values between any time point and the baseline in the placebo session from similarly calculated differences in values in the apomorphine session. Spearman's correlation was applied to examine possible associations between the apomorphine effects and the adverse effects. A Chi-Square test was applied in order to compare the genotype distribution between subjects who reached the maximum 180 sec of tolerance at baseline versus all other subjects. Since the second analysis included only one measure (tolerance), the results of this analysis were considered significant at the p<0.05 level.

The third set of analyses was conducted in order to assess the genotype differences in adverse effects by using the Kruskal Wallis Test with post hoc Mann-Whitney test. There were three common adverse effects found to be caused by apomorphine. Thus, Bonferroni correction was adjusted to three comparisons, and the results of the adverse effects analysis were considered significant at the p<0.016 level (0.05/3).

## Results

### Subjects

One hundred and five subjects were enrolled in the study. One subject quit the experiment due to severe nausea and sleepiness. Thus, in the first screening analysis, in which drug effects on the experimental pain measures were assessed, 104 subjects were included. In the second and third sets of analyses (see statistical analysis, method section), four subjects who were carriers of rare genotypes were excluded. In addition, two 9/9 carriers were excluded because they demonstrated an exceptionally large tolerance difference between the drug and the placebo at baseline (see section genotype distribution). Thus, in all genetic related analyses 98 subjects were included.

### Apomorphine effects

A comparison of all pain measures between the baseline sessions of apomorphine and the placebo revealed no significant differences (Wilcoxon tests; N.S.). The screening analysis showed that cold pain tolerance was the only pain measure affected by the apomorphine administration (Friedman test, Chi-Square = 60.21; p<0.001; Table 1).

**Table 1:** Apomorphine effects on pain measures.

Pain Phenotype	Condition	Session mean±SD (median, interquartile range)			
		Baseline	Time point 1	Time point 2	Time point 3
**Cold pressor test (CPT)**					
Threshold (sec)	apomorphine	7.1±5.3 (6, 5.5)	6.3±4.5 (5, 5.8)	7.6±7.8 (6, 5.3)	7.4±5.2 (6, 6)
	placebo	6.8±4.0 (6, 4)	7.4±6.7 (6, 4)	7.0±5.4 (5, 6)	7.1±5.1 (6, 5)
Intensity (0–100)	apomorphine	88.8±15 (95, 16)	87.9±18 (95, 16)	88.3±17 (95, 15)	88.5±17 (95, 15)
	placebo	90.0±15 (95, 10)	89.7±16 (95, 10)	89.5±16 (95, 11)	88.9±17 (95, 15)
Tolerance (sec)	apomorphine	**61.4±56** (42, 54)	52.6±54* (30, 39)	62.5±60 (38.5, 54)	70.3±63* (41.5, 106)
	placebo	60.0±56 (38, 45)	**60.4±59** (37, 54)	59.6±59 (35, 45)	**62.2±59** (38, 50)
**Thermal pain threshold (TSA)**					
Heat pain threshold (°C)	apomorphine	44.6±5.1 (45.5, 5.2)	44.6±3.8 (45.2, 4.3)	44.8±3 (45.3, 3.4)	44.6±3 (45, 4.1)
	placebo	45.3±2.7 (45.8, 3.9)	44.7±3 (44.9, 4.5)	44.8±2.7 (45, 4.2)	44.7±2.7 (44.9, 4.1)
Cold pain threshold (°C)	apomorphine	13.6±5.3 (12.9, 8)	14.2±6.2 (13.1, 9.2)	12.8±6 (12.2, 9.9)	12.5±6.2 (12.2, 9.3)
	placebo	14.0±5.4 (13.8, 8.8)	13.5±5.7 (14.1, 9.3)	12.9±5.6 (13.2, 8.5)	13.0±5.8 (13.1, 7.8)
**Heat pain intensity (TSA)**					
Intensity (0–100)	apomorphine	44.5±28 (40, 50)	48.6±26 (45, 40)	46.1±28 (40, 45)	46.2±29 (41, 45)
	placebo	45.5±27 (45, 45)	47.5±29 (45, 51)	49.5±29 (47, 54)	47.8±28 (42, 45)

Mean±SD **(median, interquartile range)** of pain phenotypes for the two study conditions in each study session are described. Asterisk (*) represents significant differences (Wilcoxon Signed Ranks Test, p<0.01) compared to baseline and placebo.

**Figure 2:**
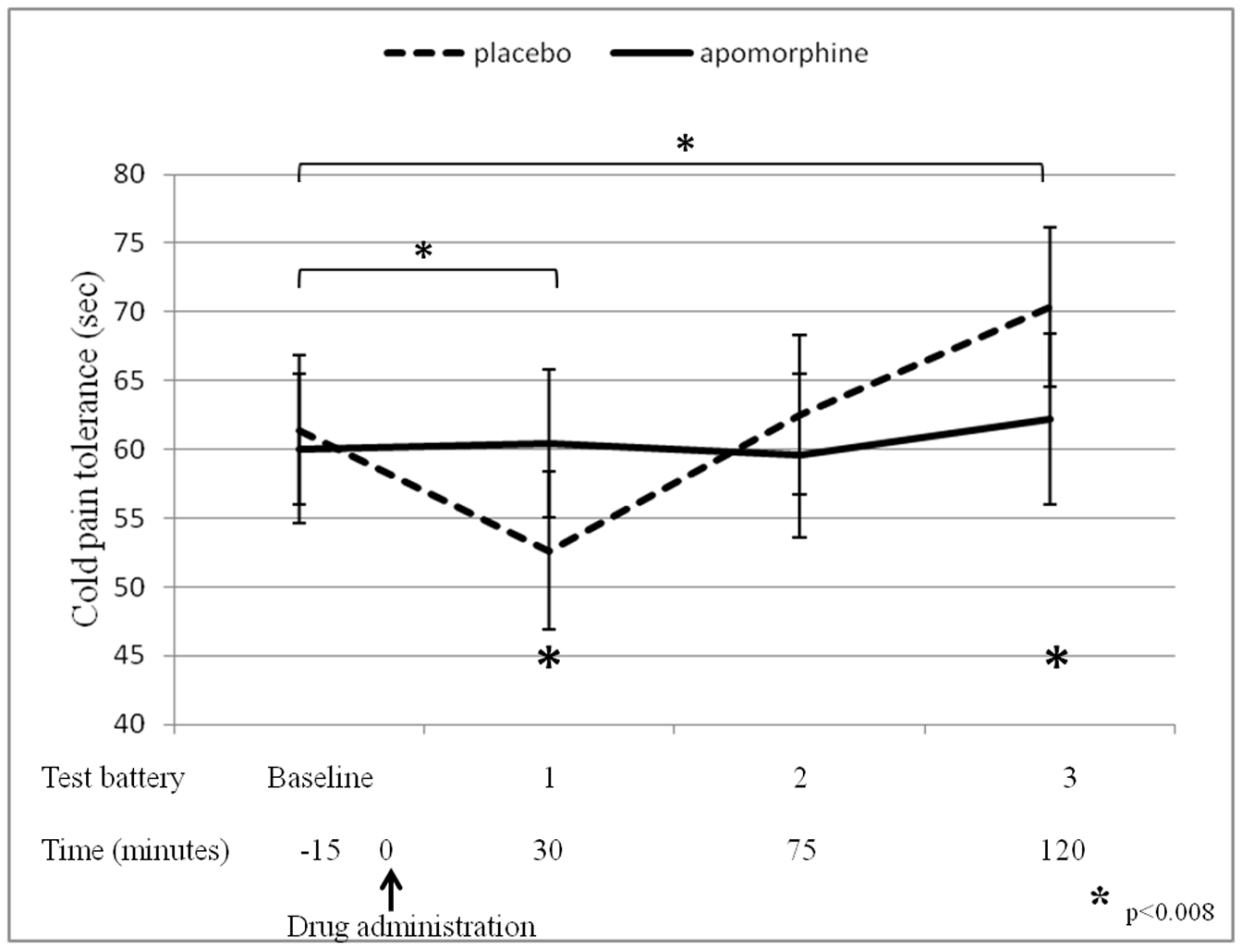
Apomorphine effect on cold pain tolerance (n = 104). Mean±SEM of cold pain tolerance (sec) for the two study conditions, measured before (baseline) and after drug administration. Upper asterisks represent significant differences between apomorphine and baseline measurements; bottom asterisks represent significant differences between apomorphine and placebo measurements. Notably, tolerance was tested 20 minutes after the initiation of each test battery.

Apomorphine induced a significant *decrease* in cold pain tolerance 30 minutes after its administration, as compared to both baseline tolerance (Wilcoxon test, Z = −4.90; Effect size = 0.48; p<0.001; 14% of tolerance decrease) and the tolerance 30 minutes following placebo administration (Wilcoxon test, Z = −3.54; Effect size = 0.34; p<0.001; 13% of tolerance decrease; Figure 2). As expected, no effects on tolerance were observed following placebo administration (Friedman test, Chi-Square = 6.39; p = 0.094). No changes in tolerance as compared to its values both at baseline and in response to placebo administration were observed at the 75 min time point.

**Figure 3:**
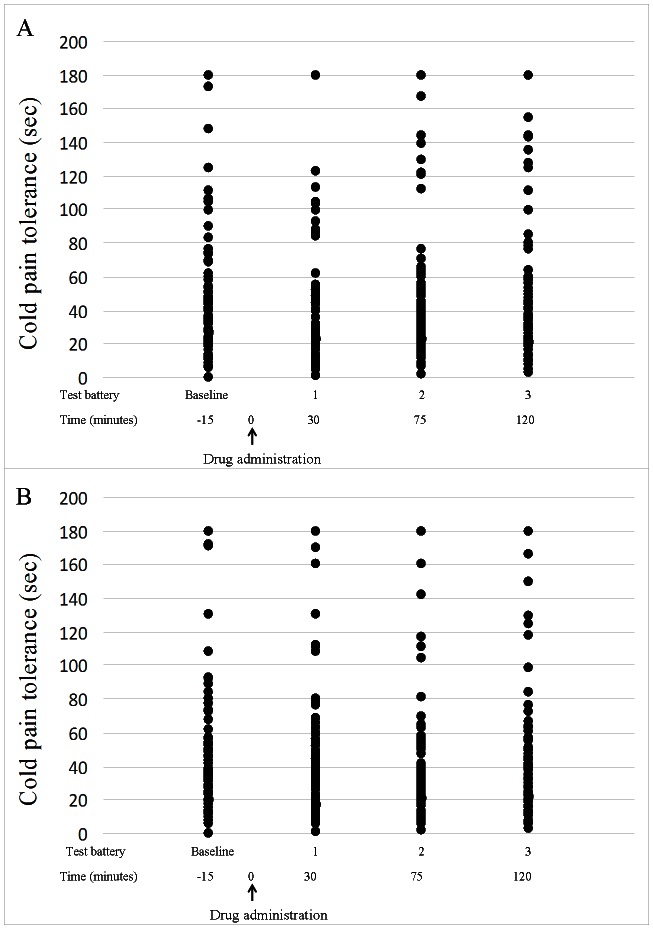
Distribution of cold pain tolerance (n = 104). Scattered plots of cold pain tolerance (sec) in the apomorphine (A) and placebo (B) conditions, measured before (baseline) and after drug administration.

A significant *increase* in tolerance 120 minutes after drug administration, as compared to both baseline tolerance (Wilcoxon test, Z = −2.94; Effect size = 0.25; p = 0.004; 15% of tolerance prolongation) and the tolerance 120 minutes following placebo administration (Wilcoxon test, Z = −3.12; Effect size = 0.31; p<0.001; 14% of tolerance prolongation; Figure 2). Notably, a non-parametric statistical approach was used for data analyses due to the abnormal distribution of tolerance (Figure 3).

### Genotype distribution

Dopamine transporter *DAT-1* allele and genotype distribution are presented in Table 2. All 105 subjects were successfully genotyped. As expected, the most common alleles were 9 and 10, with frequencies of 40% and 58%, respectively. Allele 7 had a frequency of 0.5%, and allele 11 had a frequency of 1.5%. Genotype distributions were: homozygote 9/9, 15.2%; heterozygote 9/10, 45.7%; and homozygote 10/10, 35.2%. As mentioned earlier, six subjects were excluded from all genetic related analyses: four of them because of carrying the rare genotypes 7/9 and 9/11; two additional subjects (both were 9/9 carriers) were excluded because they demonstrated an exceptionally large difference in tolerance (>20 sec) between the drug and the placebo at baseline. Thus, 98 subjects were included in the genetic analysis. The *DAT-1* polymorphism met the Hardy–Weinberg equilibrium test (95% confidence interval, p = 0.947).

**Table 2:** Dopamine transporter *DAT-1* allele and genotype distribution.

Allele	Count	Genotype	Count
7	1	7/9	1
9	84	9/9	16
10	122	9/10	48
11	3	9/11	3
		10/10	37
Total	210	Total	105

Values indicate the occurrence of each allele/genotype.

Self-reported ethnicities were as follows: 64% Ashkenazi Jews (Eastern European origin); 14% Sephardic Jews (North African/Asian origin); 10% mixed Ashkenazi and Sephardic Jews; and 12% Arabs. Given this heterogeneity, it was critical to establish whether genetic effects could have been due to occult stratification. To this end, we reanalyzed the data, omitting the minor frequency subgroups (the 14% Sephardic Jews, the 10% mixed Ashkenazi and Sephardic Jews, or the 12% Arabs) in each run. Similar patterns of results to those found for the entire study population were found in these analyses.

### Associations between the dopamine transporter polymorphism and the apomorphine effects

Since all genotype related analyses were based on comparisons of changes in values from baseline, a comparison of baseline values between the two sessions was conducted separately for each genotype subgroup. Significant differences were found only in baseline values in the 9/9 carriers subgroup (Wilcoxon test, Z = −2.48; Effect size = 0.62; p = 0.013). Upon careful examination, two outliers were identified, which demonstrated exceptionally large (72- and 24-second) differences between baseline tolerance measurements. Following the exclusion of these two, no significant differences between baseline tolerance values were found in the 9/9 carriers subgroup (Wilcoxon test, Z = −1.41; Effect size = 0.38; p = 0.076). In addition, a comparison of baseline tolerance was conducted between the three subgroups within each session and revealed no differences (Kruskal Wallis Test, Chi-square = 1.915, p = 0.384; Chi-square = 0.805, p = 0.669 in the apomorphine and the placebo conditions, respectively).

**Figure 4:**
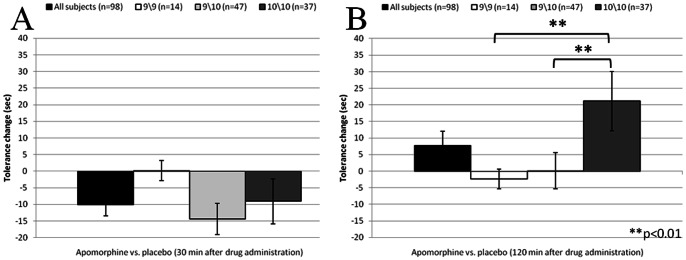
Changes in pain tolerance as compared to placebo by genotype subgroups (n = 98). (A) Changes in pain tolerance 30 minutes after drug administration; (B) Changes in pain tolerance 120 minutes after drug administration. Black bars represent drug effect across all subjects. Negative change indicates shortening in tolerance.

Despite the significant decrease in cold pain tolerance 30 minutes after apomorphine administration, as compared to both the baseline and the placebo conditions, no significant genotype differences were found in regard to this effect of apomorphine (Kruskal Wallis Tests, Chi-square = 3.98, p = 0.136; Chi-square = 2.12, p = 0.346, as compared to the baseline and the placebo conditions, respectively; Figure 4A). Similarly, no significant genotype differences in tolerance were found 75 min after drug administration (Kruskal Wallis Tests, Chi-square = 5.602, p = 0.061; Chi-square = 4.059, p = 0.131, as compared to the baseline and the placebo conditions, respectively).

Significant differences were found in the *increasing* effect of apomorphine on tolerance 120 min after drug administration, as compared to the baseline, between the dopamine transporter genotype groups (Kruskal Wallis Test, Chi-square = 6.9; p = 0.028). The homozygote carriers of the 10-allele group demonstrated longer prolongation (31% tolerance prolongation) than the homozygote carriers of the 9-allele group (2% tolerance prolongation) (Mann-Whitney U test, Z = −2.21; Effect size = 0.24; p = 0.027) and the heterozygote carriers of the 9/10 genotype (8% tolerance prolongation) (Mann-Whitney U test, Z = −2.07; Effect size = 0.23; p = 0.021). No differences in tolerance change were found in the placebo session (placebo effect) between the genotype groups 120 min after drug administration (Kruskal Wallis Test, Chi-square = 0.71; p = 0.701).

As compared to the placebo, significant differences were found in the *increasing* effect (after 120 minutes) of apomorphine between the dopamine transporter genotype groups (Kruskal Wallis Test, Chi-square = 10.27; p = 0.006). The homozygote carriers of the 10-allele group demonstrated significantly longer prolongation (35% tolerance prolongation) than the homozygote carriers of the 9-allele group (0.5% tolerance prolongation) (Mann-Whitney U test, Z = −2.81; Effect size = 0.39; p = 0.007) and the heterozygote carriers of the 9/10 genotype (1% tolerance prolongation) (Mann-Whitney U test, Z = −2.97; Effect size = 0.32; p = 0.003; Figure 4B).

Fourteen subjects reached the maximum 180 sec of tolerance (cutoff time to hand withdrawal from the cold water) at the baseline measurement prior to drug administration and therefore deserved a special consideration. A separate analysis of these subjects revealed that they significantly differed in their dopamine transporter genotype profiles (Chi-Square test, Chi-square = 10.94, p = 0.004). None of the 14 subjects were 10/10 homozygote carriers, while 11 of the 14 were heterozygote carriers. Given that these 14 subjects demonstrated a ceiling effect, meaning that no tolerance prolongation could be observed, all analyses for both drug and genotype effects were repeated without them. The results of these tests (not shown) were very similar, with all remaining significant.

### Adverse effects

Ten minutes following apomorphine administration (prior to the first pain test battery), 60% of the subjects reported at least one adverse effect, while 32% reported at least one adverse effect following placebo administration. These percentages dropped to 26% and 29%, respectively, at the 100-minute time point (prior to the last pain test battery). The most common adverse effects caused by apomorphine were sleepiness, nausea, and sweating. All adverse events resolved spontaneously, and no serious adverse effects were noted.

No significant correlations were found between any of the adverse effects and the prolonging or shortening effects of apomorphine on the tolerance to cold pain (Spearman's correlation, N.S.). The adverse side effects exhibited by the different genetic groups ten minutes following apomorphine administration (time of peak prevalence) are presented in Table 3. A significant association was found between the *DAT*-*1* polymorphism and the self-reported adverse effect score for “sweating” (Kruskal Wallis Test, Chi-square = 8.569; p = 0.014). Heterozygote carriers reported significantly higher sweating scores than the homozygote carriers of the 10-allele group (Mann-Whitney U test, Z = −2.54; Effect size = 0.28; p = 0.011). No significant differences were found between the heterozygote carriers and the homozygote carriers of the 9-allele group (p = 0.072) or between the two homozygote groups (p = 0.964). No other significant associations between the *DAT-1* polymorphism and any of the adverse effects induced by apomorphine or the placebo were found ten minutes following apomorphine administration.

**Table 3:** Adverse side effects exhibited by the different *DAT-1* genotype groups.

Adverse effect	Condition	Genotype			Chi-Square	P value
		9/9	9/10	10/10		
**Sweating**	apomorphine	0.11±0.4	0.39±0.7	0.08±0.4	8.569	0.014*
	placebo	none	0.05±0.2	none	2.571	0.276
**Dyspnea**	apomorphine	0.05±0.2	0.09±0.3	0.05±0.3	1.402	0.496
	placebo	none	0.05±0.2	none	2.571	0.276
**Dry mouth**	apomorphine	0.11±0.3	0.18±0.4	0.08±0.3	1.219	0.544
	placebo	0.11±0.3	0.11±0.4	0.13±0.3	0.294	0.863
**Sleepiness**	apomorphine	0.83±0.9	0.95±0.9	0.89±0.7	0.168	0.920
	placebo	0.28±0.6	0.44±0.6	0.34±0.5	1.275	0.529
**Headache**	apomorphine	0.22±0.7	0.21±0.5	0.14±0.4	1.023	0.600
	placebo	none	0.16±0.4	0.05±0.2	3.989	0.136
**Nausea**	apomorphine	0.33±0.7	0.54±0.8	0.35±0.8	2.748	0.253
	placebo	none	0.06±0.3	0.11±0.3	2.040	0.361
**Confusion**	apomorphine	0.11±0.3	0.23±0.5	0.19±0.6	0.981	0.612
	placebo	0.11±0.3	0.23±0.5	0.19±0.6	1.227	0.541

Mean ±SD of the reported adverse effects in the two study conditions ten minutes following apomorphine administration are described. The Chi-Square and P value columns illustrate the Kruskal Wallis test results. Median values are not described given that they all equal 0.

## Discussion

The main finding of the present study was that the administration of the nonspecific dopamine agonist apomorphine had an effect on cold pain tolerance, but it did not have any effect on thermal pain threshold or intensity. Moreover, an association was found between the *DAT-1* gene polymorphism and the prolonging effect of apomorphine on cold pain tolerance.

The fact that only tolerance to cold pain was affected by apomorphine deserves consideration. First, it is in line with the results of a previous study conducted in our laboratory, in which associations were found between dopamine-related gene polymorphisms and cold pain tolerance, but not with perceived pain intensity in response to either heat or cold stimuli [6]. At the same time, the lack of effect of apomorphine on thermal pain thresholds or intensities in our study seems to contradict the results of some other studies demonstrating that dopaminergic manipulations did have an effect on pain threshold and intensity in painful clinical conditions, such as burning mouth syndrome, fibromyalgia, Parkinson's disease, and restless leg syndrome [16]–[21]. However, other studies failed to show the effect of dopaminergic interventions on these parameters. One example is a recent study in which apomorphine administration was not found to have an effect on either objective or subjective pain thresholds in patients with Parkinson's disease. It should be noted that pain tolerance was not assessed in that study [22]. Therefore, it is clear that the effects of dopamine on pain are poorly understood. Second, tolerance is known to indicate the degree of willingness to continue enduring an intense stimulus and is largely influenced by motivation [23]. At the same time, motivation has been shown to be closely associated with dopamine activity [24], [25]. Neuroanatomically, this may suggest that the ventral tegmental area, a dopaminergic pathway known to be related to motivation and effort-related functions [26], [27], is likely involved in the observed change in tolerance to cold pain following apomorphine administration. Lastly, given the wide effects of dopamine on many functions such as motivation and attention, further research is needed in order to investigate if the effects found in the present study are a direct effect of apomorphine on nociception, or a part of a broader, non-specific effect on high mental functions.

As shown in the results, apomorphine had a complex effect on pain tolerance, which consisted of an initial tolerance shortening effect followed by an opposing tolerance prolonging effect. Although we have no clear explanation for this complex effect, several explanations should be considered: (1) The “U shape theory” according to which pain is affected by direct activation of post-synaptic dopaminergic receptors by apomorphine. In this case, the complex effect can be explained by the pharmacokinetics of apomorphine, which peaks shortly after its administration, but has a rather short half-life and by that creates an “inverted U behavioral curve”. “Inverted U” relationships have been demonstrated recently in relation to dopaminergic activity and high cognitive functions [28]. This phenomenon can be explained by a potential initial shift all subjects, regardless of their genetic predisposition, to the right end of dopamine activity continuum, thus reducing their ability to tolerate pain. This is then followed by a decline in dopaminergic activity to an “optimal” analgesic phase. Similar biphasic dose dependent effects of apomorphine [29] and L-dopa [30] on nociceptive behavior in rodents have been reported, thus indicating that a lower dose of apomorphine might have abolished the hyperalgesic phase found in our study.

(2) The “tonic-phasic dopaminergic activity theory”. According to this theory phasic dopamine system is activated by brief bursts of neuronal firing, while tonic dopamine activity refers to the level of extrasynaptic dopamine [31]. At the same time, high tonic dopamine attenuates phasic dopamine release whereas low tonic dopamine facilitates phasic dopamine firing [32]. It has been hypotheses that any analgesic effects of dopamine rely on the phasic dopamine system [33]. Apomorphine administration causes a temporal increase in tonic dopamine levels and by that significantly inhibits phasic dopamine secretion [34]. Hence, it can be assumed that the early hyperalgesic phase results from the increase inhibition of phasic dopamine signaling duo to the high tonic doaminergic activity 30 min after apomorphine administration. In contrast, 120 min after its administration, apomorphine is still present, although to a much lesser extent, therefore reduces the tonic inhibition and allows an increase in the phasic dopaminergic analgesic firing [35].

(3) Another possible explanation of the complex pain-dopamine interaction relates specifically to apomorphine in several ways. First, the exact location in the CNS where the effects of apomorphine on pain occur is not yet known. While at least one animal study suggests that the apomorphine antinociceptive effect can be mediated by activating dopamine receptors in the ventrolateral orbital cortex [36], another study points to the periaqueductal gray (PAG) as the site of apomorphine induced anti-nociception [37]. Second, apomorphine is a non-specific dopamine agonist, which also interacts with other catecholamine receptors [13] and therefore activates a variety of both pain inhibiting and pain facilitating mechanisms. Future studies aimed to test different doses of apomorphine and other dopamine agonists are required for better understanding of these complex dopamine-pain interactions.

The functionality of *DAT-1* VNTR has been under debate in the literature and remains controversial [38], [39]. While some studies have shown no associations between DAT-1 VNTR dopamine availability, other studies have demonstrated the presence of such association, although in different directions: VanDyck et al. [40] reported increased dopamine transporter availability associated with the 9-repeat allele of the *DAT-1* VNTR. In contrast, both in vitro [41] and in vivo [42] studies have shown that the 10-repeat allele produces significantly higher levels of dopamine transporter than the 9-repeat allele. Thus, the 10-repeat allele presumably leads to relatively decreased extra-synaptic dopamine levels. In line with the latter two studies, homozygote carriers of the 10-allele in our study demonstrated high tolerance prolongation (31%–35% tolerance prolongation) following apomorphine administration. Hence, our results support the notion according to which homozygote carriers of the 10-allele are predisposed to relatively low dopamine availability, and therefore are more susceptible to apomorphine. Nonetheless, additional studies are needed in order to further explore DAT-1 VNTR functionality.

Notably, previous studies have shown associations between the effect of dopamine agonists on the performance of cognitive tasks and dopamine-related gene polymorphisms [43], [44]. To the best of our knowledge, this is the first demonstration of a similar association in relation to pain. The fact that none of the 14 subjects who demonstrated a maximal tolerance to cold pain at baseline was a 10/10 homozygote carrier provides support for our previous results [6], demonstrating that 10/10 carriers are less capable of tolerating pain.

Other functional polymorphisms within dopamine-related genes are known to be associated with different pain-related phenotypes [7]–[11]. In our previous work, polymorphisms in both the *DAT-1* and *MAO-A* genes were found to be associated with tolerance to cold pain [6]. However, given that the *MAO-A* gene is located on the X chromosome, its effect can be assessed only in women, thereby dramatically reducing its study power. Thus, the current work was focused only on the *DAT-1* genotype. Future studies using larger cohorts can potentially reveal any associations between other dopamine-related gene polymorphisms and the response to dopaminergic treatments.

Several limitations of the present study deserve consideration: First, there were differences in the prevalence of some side effects in response to the placebo as compared to the active treatment. This may have eliminated the blind aspect of the study by revealing the true nature of each group. Yet, the fact that no correlations were found between any of the adverse effects and the effects of apomorphine on tolerance implies that it is unlikely the adverse effects skewed the results. The use of an active placebo that mimics the adverse effect profile of the tested drug can resolve this limitation in future studies. Second, apomorphine might have had an effect on skin blood flow, which could hypothetically alter the response to the cold pain stimuli. However, in such a case it would be difficult to explain that fact that tolerance was the only cold pain parameter affected. Third, no quality control for genotypes was done by replication of the genotype analysis. However, in earlier studies conducted in our laboratory, such replications yielded error rates of less than 1%. This magnitude of error is unlikely to have any effect on the results of the present study. Lastly, there is evidence for involvement of peripheral dopamine receptors in nociception [45], [46]. Therefore theoretically, such peripheral effects of apomorphine could have contributed to the changes in pain tolerance found in our study. However, those effects were expected to be blocked by the pre-administration of the peripheral dopamine antagonist domperidon before placebo and apomorphine. One can therefore argue that all peripheral dopaminergic effects have been eliminated in the two conditions and only the central effects of apomorphine (against placebo) has been tested. Conversely, by blocking peripheral dopaminergic receptors, domperidone by itself might have altered baseline tolerance. This possibility cannot be ruled out until studied in a separate randomized controlled trial.

In conclusion, the current study demonstrated for the first time an effect of dopamine agonist administration on cold pain tolerance in healthy subjects. The prolonging effect of apomorphine on pain tolerance was genetically determined. In addition, apomorphine produced a non-genetically associated tolerance-reducing effect. In light of this phenomenon, a search for other dopaminergic agents capable of enhancing, while not reducing, the ability to tolerate clinical pain is warranted.

## Supporting Information

Table S1 **TREND Checklist.**
(PDF)
